# Screening of genetic variants in *ADCYAP1R1*, *MME* and *14q21* in a Swedish cluster headache cohort

**DOI:** 10.1186/s10194-017-0798-y

**Published:** 2017-08-22

**Authors:** Caroline Ran, Carmen Fourier, Julia M. Michalska, Anna Steinberg, Christina Sjöstrand, Elisabet Waldenlind, Andrea Carmine Belin

**Affiliations:** 1 Department of Neuroscience, Karolinska Institutet, 171 77 Stockholm, Sweden; 2 Department of Clinical Neuroscience, Karolinska University Hospital, Stockholm, Sweden

**Keywords:** Ch, Trigeminal autonomic cephalalgia, Neprilysin, PACAP receptor, Association, SNP

## Abstract

**Background:**

We have genotyped a Swedish cluster headache case-control population for three genetic variants representing the most significant markers identified in a recently published genome wide association study on cluster headache. The genetic variants were two common polymorphisms; *rs12668955* in *ADCYAP1R1* (adenylate cyclase activating polypeptide 1 receptor type 1), *rs1006417*, an intergenic variant on chromosome 14q21 and one rare mutation, *rs147564881*, in *MME* (membrane metalloendopeptidase).

**Results:**

We screened 542 cluster headache patients and 581 controls using TaqMan real-time PCR on a 7500 fast cycler, and pyrosequencing on a PSQ 96 System. Statistical analysis for genotype and allele association showed that neither of the two common variants, *rs12668955* and *rs1006417* were associated with cluster headache. The *MME* mutation was investigated with pyrosequencing in patients, of whom all were wild type.

**Conclusion:**

In conclusion *rs12668955* and *rs1006417* do not impact the risk of developing cluster headache in the Swedish population. Also, *rs147564881* does not seem to be enriched within the Swedish cluster headache patient group.

## Background

Cluster headache (CH) is a severe primary headache affecting around 0.1-0.2% of the Swedish population [9]. There are no known causes of CH today, but a genetic component in the etiology is suggested. For instance, having a first or second degree relative with CH implies an increased risk of developing CH [4]. Twin studies also provide an indication of genetic influence in CH. Apart from case-reports identifying occasional monozygotic twins with CH, Ekbom et al. reported two concordant monozygotic twin pairs in a population of over 30,000 individuals in an interview-based register study from 2006, indicating that there is a weak heritability for CH [6, 9, 11, 16, 18]. In addition, there have been several reports of genetic associations linking genetic variations in several candidate genes to CH. Hypocretin receptor 2 (*HCRTR2*) and nitric oxide synthase (*NOS*) are examples of such genes, representing a link between the molecular pathways involved in the pathophysiology, and genetic risk-factors [3, 14, 19]. *HCRTR2* in particular has received a lot of attention due to its involvement in the regulation of sleep and pain, functions highly relevant in the CH pathophysiology [5]. Moreover, hypocretin-1 levels are reported to be reduced in the ceresbrospinal fluid in CH patients [2]. Recently the first genome wide association study (GWAS) on a CH cohort was published. Though underpowered, the GWAS data indicated a few genetic markers potentially of interest for CH pathophysiology which warrant more thorough investigation [1].

We selected the top three associations from Bacchelli et al.; *rs1006417*, an intergenic variant on chromosome 14q21; *rs12668955* in adenylate cyclase activating polypeptide 1 receptor type 1 (*ADCYAP1R1*) and one rare mutation, *rs147564881*, in the membrane metallo-endopeptidase gene (*MME*), and performed a replication study on a well characterized and large Swedish CH case-control population. Replicating GWAS findings in independent materials is a crucial step in the validation of GWASs. In particular when the discovery cohort is small, it is important to verify the association and evaluate the importance of the identified markers in patients from different genetic backgrounds. *ADCYAP1R1* and *MME* are attractive candidate genes since they are both involved in pain signaling, and our study may shed more light on the possibility of these genetic markers being involved in the pathophysiology of CH.

## Methods

We genotyped 542 CH patients and 581 controls for two single nucleotide polymorphisms (SNPs); *rs12668955* and *rs1006417*, and one rare mutation; *rs147564881*. All experiments were approved by the regional ethical review board in Stockholm, Sweden. CH patients were recruited after informed consent at the Neurology Department at Karolinska University Hospital, or through collaboration with neurologists at other clinics in the central part of Sweden. Diagnosis was confirmed by a neurologist and complied with the guidelines of the 3rd version of the International Classification of Headache Disorders (ICHD III) [20]. Patients were requested to provide personal, clinical and lifestyle information through a questionnaire and give a blood sample. Of the CH patients 31.7% were female, average age 52 years, median age 53 years, 10.3% had chronic CH, age of onset was 31.6 years and 10.8% reported they had at least one first or second degree relative with CH (53 patients of 489 for whom the information was available). Control subjects were anonymous healthy blood donors, of whom 44% were females, between the age 18 and 65.

DNA was prepared from blood using the Gentra Puregene Blood kit according to standard protocols (QIAGEN, Hilden, Germany). Genotyping was performed with TaqMan quantitative real-time PCR (qPCR) on an ABI 7500 Fast cycler (Applied Biosystems, Foster City, CA, USA). We used pre-made TaqMan SNP genotyping assays, C__32158964_10 for *rs12668955* and C__2056560_10 for *rs1006417*, and TaqMan genotyping master mix (Applied Biosystems).

TaqMan genotyping was unsuccessful for the rare mutation *rs147564881*, therefore we used pyrosequencing to analyze the *MME* mutation. We used primers (Thermo Fisher Scientific, Waltham, MA USA) designed in-house using primer3 and mfold software, as well as the NCBI online Blast tool (https://blast.ncbi.nlm.nih.gov/Blast.cgi), sequence available on request [10, 21, 22]. The genomic region of interest was amplified with a touchdown PCR spanning from 45 to 55 °C using one regular primer and one biotinylated primer. The PCR fragments were denatured and the biotin containing fragments were annealed to streptavidin-sepharose beads using a PyroMark Vacuum Prep tool (Biotage AB, Uppsala, Sweden), washed and incubated with the pyrosequencing primer at 80 °C for 2 min, and then analysed in an automated pyrosequencing PSQ 96 System using PyroMark Gold reagents (QIAGEN).

Chi-squared (χ^2^) test was used for statistical analysis using GraphPad Prism v5.04 (GraphPad Softwares Inc., La Jolla, CA, USA). Fisher’s exact test was used for the additional dominant genotypic model. Both SNPs were tested for Hardy Weinberg equilibrium (HWE) using the Online Encyclopedia for Genetic Epidemiology studies HWE calculator [15].

## Results

We genotyped 542 CH patients and 581 control subjects for two SNPs, *rs12668955* and *rs1006417* that were recently suggested to confer decreased risk for CH in a GWAS. Both SNPs were in HWE in both cases and controls (data not shown). *rs12668955* genotype and allele frequencies were similarly distributed between cases and controls. The wild-type genotype and allele of *rs1006417* appeared to be more common in the control group as compared to CH patients, however this was not reflected by the statistical analysis (Table 1). Genotype and allele analysis with χ^2^ revealed that none of the SNPs were associated with CH (Table 1). Since the genotype frequencies differed slightly between cases and controls for *rs1006417*, we further analyzed this SNP under a dominant genotypic model (AA vs. AG + GG) using a Fisher’s exact test. This analysis was consistent with the basic χ^2^ test and showed no significant association between *rs1006417* and CH (odds ratio 1.21, 95% confidence interval of 0.95-1.54 and a *p*-value of 0.12). We also investigated the presence of the rare mutation *rs147564881* in the *MME* gene in our CH population. The genomic region was difficult to analyze because of low guanine-cytosine content. Using pyrosequencing we acquired a readable sequence for 492 individuals (91% of the patients), all of whom were wild-type. Because of the lack of mutation carriers in the CH patients we did not proceed with genotyping of the controls.

**Table 1:** Results from the genotype and allele analysis of *rs12668955* and *rs1006417*

SNP	Genotype/Allele	Controls % (n)	CH % (n)	χ^2^ (df)	*p*-value
*rs12668955*	GG	30.6 (172)	31.8 (172)	0.22 (2)	0.90
	GA	48.4 (272)	47.1 (255)		
	AA	21.0 (118)	21.1 (114)		
	G	54.8 (616)	55.4 (599)	0.05 (1)	0.83
	A	45.2 (508)	44.6 (483)		
*rs1006417*	AA	62.3 (354)	57.8 (309)	2.48 (2)	0.29
	AG	32.0 (182)	35.5 (190)		
	GG	5.6 (32)	6.7 (36)		
	A	78.3 (890)	75.5 (808)	2.33 (1)	0.13
	G	21.6 (246)	24.5 (262)		

*SNP* single nucleotide polymorphism, *n* number, *CH* cluster headache, *χ* ^*2*^ Chi-square, *df* degrees of freedom

## Discussion

We have performed a replication study based on the recent findings of the first published GWAS on CH. The three most significant variants, *rs12668955*, *rs1006417* and *rs147564881* were selected and genotyped in a Swedish CH case-control study population. We found no association for either genotypes or alleles with CH for the common SNPs, and further we found no CH patient carrying the mutated allele of *rs147564881*. The discrepancy between our study and the discovery study can have several reasons. The control materials exhibit considerable differences. The Italian GWAS used 360 cigarette smokers, while we used anonymous blood donors, 14% of which are presumably smokers, as this is the proportion of smokers in the Swedish population. Moreover, there is a significant difference in gender ratio between the Swedish and the Italian material. The Italian cohort have an unusually high proportion of males (84%) compared to the Swedish cohort (68,3%). As a control experiment, we therefore verified our results in a gender stratified analysis conducted with male subjects only, successfully replicating a lack of association. Also, there might be differences in genetic background between the Swedish and Italian populations. The minor allele frequencies (MAF) for the two common SNPs differs between our study and the Italian report, in particular for *rs1006417* which is protective in the Italian study. In our material, although the difference is non-significant, cases have a higher MAF (0.245) than controls (MAF 0.216) which would be indicative of an increased rather than decreased risk. The association discovered in the Italian cohort might also reflect a linkage disequilibrium (LD) between these SNPs and other genetic variations truly linked to disease that are not present in the Swedish population. Since we did not genotype any additional marker at these loci, we could not control for a potential difference in the LD structure between the Swedish and Italian populations. Another limitation of our study is the possibility that other rare *MME* mutations might be associated to CH in our material. Last, life style and environmental factors, e.g. seasonal variation has been reported to influence CH, smoking is a risk factor for CH, other plausible factors might be inflammations and diet, [7, 8, 12, 13, 17], and gene-environment interactions might cause genetic risk factors to differ in specific populations or various parts of the world.

## Conclusion

In conclusion *rs12668955* and *rs1006417* do not impact the risk of developing CH in the Swedish population. Also, *rs147564881* is a rare mutation which does not seem to be enriched within the Swedish CH patient group.

## Abbreviations

ADCYAP1R1: Adenylate cyclase activating polypeptide 1 receptor type 1
CH: Cluster headache
df: Degrees of freedom
GWAS: Genome wide association study
HCRTR2: Hypocretin receptor 2
HWE: Hardy Weinberg equilibrium
ICHD: International Classification of Headache Disorders
IHS: The Iternational Headache Society
MAF: Minor allele frequencies
MME: Membrane metalloendopeptidase
NOS: Nitric oxide synthase
qPCR: Quantitative real-time PCR
SNP: Single nucleotide polymorphisms
χ^2^: Chi-square
